# Evaluation of a Previously Suggested Plasma Biomarker Panel to Identify Alzheimer's Disease

**DOI:** 10.1371/journal.pone.0029868

**Published:** 2012-01-18

**Authors:** Maria Björkqvist, Mattias Ohlsson, Lennart Minthon, Oskar Hansson

**Affiliations:** 1 Brain Disease Biomarker Unit, Department of Experimental Medical Science, Wallenberg Neuroscience Center, Lund University, Lund, Sweden; 2 Computational Biology and Biological Physics, Lund University, Lund, Sweden; 3 Clinical Memory Research Unit, Department of Clinical Sciences Malmö, Lund University, Malmö, Sweden; 4 Neuropsychiatric Clinic, Skåne University Hospital, Malmö, Sweden; Virginia Commonwealth University, United States of America

## Abstract

There is an urgent need for biomarkers in plasma to identify Alzheimer's disease (AD). It has previously been shown that a signature of 18 plasma proteins can identify AD during pre-dementia and dementia stages (Ray et al, Nature Medicine, 2007). We quantified the same 18 proteins in plasma from 174 controls, 142 patients with AD, and 88 patients with other dementias. Only three of these proteins (EGF, PDG-BB and MIP-1δ) differed significantly in plasma between controls and AD. The 18 proteins could classify patients with AD from controls with low diagnostic precision (area under the ROC curve was 63%). Moreover, they could not distinguish AD from other dementias. In conclusion, independent validation of results is important in explorative biomarker studies.

## Introduction

Alzheimer's disease (AD) is the major cause of dementia and a great medical and socioeconomic problem worldwide. As populations get older, the prevalence of AD will increase considerably during the coming decades [1]. The pathological characteristics of AD are senile plaques and neurofibrillary tangles, containing aggregated amyloid β (Aβ) and hyperphosphorylated tau protein, respectively [1], [2]. Aβ accumulation is thought to start many decades before symptoms occur [3]. During the last few years, it has become more apparent that disease-modifying therapies for AD are more likely to be successful if initiated during the early stages of the disease when neurodegeneration is not yet too severe [4], [5]. Therefore, biomarkers are urgently needed to correctly identify subjects affected by AD before they have developed dementia [5], [6]. Cerebrospinal fluid biomarker can identify prodromal AD with acceptable accuracy [7]–[9]. However, plasma is much easier obtained than cerebrospinal fluid. Therefore, it was a major breakthrough when Ray and collaborators found that a pattern of 18 proteins in plasma could classify samples from AD and controls with almost 90% accuracy [10]. The same plasma proteins could also predict the patients with mild cognitive impairment who would later develop AD. The study comprised of 259 plasma samples obtained from in total 7 clinical centres [10].

In the present study, we evaluated the diagnostic value of the same 18 proteins as Ray et al [10], using 433 plasma samples obtained at Skåne University Hospital, Sweden, from 174 controls, 142 patients with AD, 29 patients with depression, and 88 patients with other types of dementia than AD (i.e 37 with Lewy Body dementia, 11 with Parkinson's disease with dementia, 22 with frontotemporal dementia, 18 with vascular dementia).

## Materials and Methods

### Collection and processing of human plasma samples

The study population was recruited at the memory disorder clinic, Skåne University Hospital, Malmö, Sweden. The patients underwent thorough standard examinations conducted by a trained physician, including neurological, physical and psychiatric examinations. The patients who during clinical follow-up received a diagnosis of AD had to meet the DSM-IIIR criteria of dementia [11] and the criteria of probable AD defined by NINCDS-ADRDA [12]. Subjects who were diagnosed as having vascular dementia (VaD) fulfilled the DSM-IIIR criteria of dementia and the requirements for probable VaD by NINDS-AIREN [13] or the recommendations by Erkinjuntti and co-workers for VaD of the subcortical type [14]. For patients who developed dementia with Lewy bodies (DLB) or frontotemporal dementia, the consensus criteria by McKeith and collaborators [15] and McKhann and colleagues were used [16], respectively. The healthy volunteers had no memory complaints or other cognitive symptoms, preservation of general cognitive function, and no active neurological or psychiatric diseases.

The study was conducted in accordance with the Helsinki Declaration and approved by the ethics committee of Lund University, Sweden. All subjects gave informed written consent.

Non-fasting plasma was collected between 9 and 11 am. After venipuncture, blood was collected in tubes prepared with EDTA to prevent coagulation. Samples were centrifuged, and plasma was removed from the tubes leaving 1 ml of plasma to avoid contamination of plasma with blood cells including trombocytes. Within one hour from venipuncture the plasma was frozen in polypropylene tubes at −80°C until biochemical analysis.

### Analysis of plasma proteins

Quantibody® Human Costum Cytokine Antibody Array was performed by RayBiotech (as per company description) on blinded samples for the following markers; ANG-2I, CAM-1, IGFBP-6, PARC, PDGF-BB, RANTES, EGF, G-CSF, GDNF, IL-1a, IL-3, IL-8, IL-11, MCP-3, M-CSF, MIP-1δ, TNFa, and TRAIL R4. A positive control (four biotin-labelled bovine IgG spot) was included on each array and used for inter- and intra-slide normalization. For a quadruplicate spot, outliers as value above 30% over the median, was excluded. All samples were analyzed in a single run to minimize variation.

Selected cytokines (M-CSF and TNF-α) were quantified in triplicates using Meso Scale Discovery (MSD®, Gaithersburg, MD) electrochemoluminescence assays using a modification of the manufacturer's protocol. 30 ul was used as the sample volume and a 10-point standard curve was used, ranging from 2500 pg/ml to 0 pg/ml. The sample and calibrator were incubated on the MSD plate for 3 h (instead of 2 h), followed by a wash (as per manufacturer's recommendation). The MSD plate was then incubated with detection antibody solution for 3 h (instead of 2 h) before wash and read as per manufacturer's recommendation. Results were analyzed on a SECTOR™ 6000 instrument (MSD). The operator was unaware of the disease state of each sample during processing and statistical analysis was performed independently.

### Statistical analysis

The statistical analyses were accomplished using SPSS for Windows, version 18.0.1 (SPSS Inc/IBM, Chicago, IL, USA). To compare demographic and plasma data between groups, non-parametric Kruskal-Wallis tests were performed followed by Mann-Whitney *U*-tests for continuous variables. Pearson's *x* ^2^ test was used for dichotomous variables.

To assess the ability of the plasma data to separate groups (AD vs. Controls or AD vs. other dementia) multiple logistic regression [17], artificial neural network (ANN) [18] and nearest shrunken centroid [19] classification models were used. The latter was the method used by Ray et al. Bagging ensembles [20] of standard multi-layer perceptrons with one hidden layer were used in the ANN models. The size of the ensemble was set to 30 and the number of hidden nodes of the individual networks in the ensemble was two. No effort was made to tune these parameters. The nearest shrunken centroid method was implemented using the R package *pamr*. The area under the ROC curve (AUC) was used to measure the performance of the classification models. For all three models, 10-fold cross-validation was used to estimate true AUC values. The cross-validation procedure was repeated 100 times, with random 10-fold splits each time, in order to decrease random fluctuations.

## Results

In table 1 we present the demographic data and levels of the 18 plasma proteins obtained when using Quantibody® Human Costum Cytokine Antibody Array (RayBiotech). The subjects affected by AD were slightly older than the controls and the group affected by other forms of dementias (p≤0.01). Only three proteins of the 18 proteins, (EGF, PDG-BB and MIP-1δ), were found to be significantly altered in plasma from AD patients when compared to controls (table 1). None of the proteins differed between the AD group and the group with other dementias than AD. Analyses of two cytokines (M-CSF and TNF-a) with ELISA technology verified that there were no statistical differences between AD and control plasma samples (in control plasma, n = 148, M-CSF levels were 21.82±0.87 ng/L and TNF-a levels were 1.90±0.14 ng/L, in AD plasma, n = 148, the corresponding levels were 24.03±0.71 and 1.85±0.06 ng/L respectively).

**Table 1:** Subject demographics and plasma protein levels.

	Controls (n = 174)	Depr (n = 29)	AD (n = 142)	FTD (n = 22)	VaD (n = 18)	PDD (n = 11)	DLB (n = 37)
Mean age (range)	74 (62–99)	59 (42–76)	76^a^^,^ b (56–87)	62 (43–78)	76 (56–84)	72 (62–81)	74 (54–85)
Women	117	15	40^c^	11	14	5	26
MMSE	29±0,1	28±0,4	21±0,4^d^^,^ e	22±1,1	22±0,8	20±2,0	21±0,9
ANG-2	2444±143	1710±232	2306±284	2043±320	2410±527	1789±535	2335±341
ICAM-1	224±9	250±31	216±9	222±28	220±22	162±16	204±15
IGFBP-6	382±13	366±21	377±13	337±19	373±40	391±51	379±17
PARC (CCL18)	43±2,7	40±6,0	40±2,4	38±5,1	39±7,9	33±5,3	41±3,9
PDGF-BB	4385±288	6339±1122	5701±427^f^	5896±706	9302±3278	8557±3513	5798±1431
RANTES (CCL5)	18±0,7	20±1,5	20±0,7	20±1,3	23±3,2	19±2,3	18±1,3
EGF	541±31	665±65	706±32^g^	825±79	799±158	907±117	580±49
G-CSF	54±3,1	41±5,1	57±6	56±10	53±12	56±13	42±3,9
GDNF	103±33	86±34	322±262	79±15	41±11	34±12	45±6,5
IL-1a	13±2,4	11,2±3,2	26±17	11±1,7	8,5±1,9	10±2,0	9,5±1,3
IL-3	44±15	29±9,1	87±63	32±6,3	14±3,8	15±4,1	22±6,0
IL-8 (CXCL8)	11±1,0	8,5±0,8	10±0,5	12±1,9	10±1,4	11±1,2	10±0,8
IL-11	236±27	185±78	249±34	249±53	260±74	158±563	263±49
MCP-3 (CCL7)	51±4,8	47±6,4	82±30	51±6,8	52±12	67±8,2	42±6,1
M-CSF	1,0±0,2	1,3±0,7	1±0,3	1,2±0,37	0,77±0,51	1,2±0,5	1,0±0,4
MIP-1d (CCL15)	2361±85	2322±229	2845±142^h^	2452±200	2383±312	3154±453	3144±348
TNF-a	23±4,6	15±2,5	17±1,3	17±2,6	15±2,8	16±2,6	16±2,0
TRAILR4	36±15	12±4,3	169±157	17±7,1	13±9,1	3,1±2,3	9,5±3,3

Table 1. Values are given in ng/L, except for ICAM-1, IGFBP-6, PARC and RANTES for which concentrations are given in µg/L.

When comparing AD vs 1) Controls, 2) Depression and 3) other dementias the following significant changes were observed:

a AD vs control p = 0.01,

b AD vs depression p<0.001, AD vs other dementias p = 0.01.

c AD vs depression p = 0.034.

d AD vs control p<0.001,

e AD vs depression p = 0.001.

f AD vs control p = 0.004.

g AD vs control p<0.001.

h AD vs control p = 0.011 (Kruskal–Wallis one-way analysis of variance by ranks followed by Mann-Whitney *U* test).

Abbreviations: Depr, depression; AD, Alzheimers disease; FTD, frontotemporal lobe dementia; VaD, vascular dementia; PDD, Parkinson's disease with dementia; DLB = dementia with Lewy bodies.

When classifying the AD group from the controls, the cross-validation AUC for the logistic regression model was 0.60 using all 18 proteins. The corresponding AUC for the ANN model and the nearest shrunken centroid classifier was 0.63. When only using the three proteins that differed significantly between groups (i.e. EGF, PDG-BB and MIP-1δ), as inputs to the classifiers, the AUC increased to 0.66 for all three models.

A worse performance was obtained when classifying the group with AD from the group with other forms of dementia than AD. Using all plasma proteins the cross-validation AUC was below 0.5 indicating no classification ability at all. This was true for all three models. The best individual protein in terms of AUC performance was TRAIL-R4 with an AUC of 0.61 (cross-validation result).

**Figure 1:**
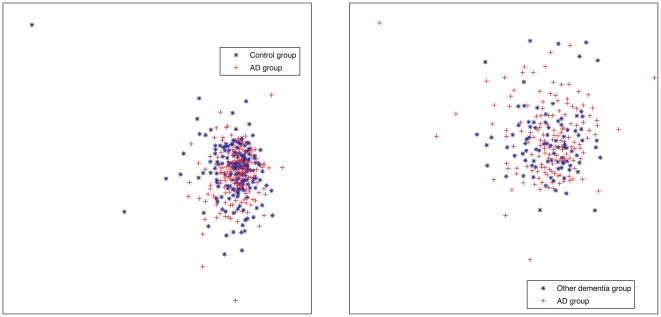
Multidimensional scaling plots (MDS) for the data. The left figure shows a MDS projection to 2 dimensions, using all 18 proteins, for the AD and the Control groups. The right figure is the corresponding plot for the AD and the other dementias group.

To further illustrate the limitation of the 18 plasma protein panel to differentiate AD from the controls and other dementia groups, multidimensional scaling (MDS) plots were produced (figure 1). These plots show a large degree of overlap between the diagnostic groups.

## Discussion

Characterizing protein markers in plasma has created optimism for finding detectable disease-specific pattern of changes. A biomarker panel of eighteen plasma proteins were shown in 2007 to classify blinded samples from AD and control subjects with close to 90% accuracy and to identify patients who had mild cognitive impairment that progressed to Alzheimer's disease [10]. The study was comprised of 259 plasma samples obtained from in total seven different clinical centres.

Interestingly, when re-analysing the same data set, originally obtained from the Ray et al study, a subset of plasma proteins (as z-scores of plasma proteins) resulted in good diagnostic accuracy [21]. However, following up on these results, using bead-based multiplex technology, Soares and co-workers have shown that when using a subset of the proteins included in the original 18 protein panel a diagnostic accuracy of only 61% was obtained when differentiating cases with AD from controls [22]. Later Rocha de Paula et al. proposed, using the original data set provided by Ray et al, that including pair-wise differences of z-score values to the mathematical method, could collectively provide a good discrimination value [23].

In the present study we found that the 18 plasma protein panel could classify samples from AD and controls with an AUC of only 63%, indicating that this protein panel cannot be used in the clinical diagnostic work-up of AD. The same protein panel could not distinguish cases with AD from subjects affected by other forms of dementia. In addition, the pattern of protein changes observed in the present study was not the same as in Ray et al. More specifically, in the training set described in the study by Ray et al [10], plasma z- levels of PDGF-BB, EGF and MIP-1δ were seen to be reduced in AD cases. In contradiction to this, in the present study the plasma levels of these proteins were increased in the AD cases. Similarly, Marksteiner et al have found that plasma MIP-1δ and EGF are increased in AD patients when compared to patients affected by depression [24].

Several of the 18 proteins included in the biomarker panel are involved in the immune response [10]. There are, however, important caveats to the use of plasma immune markers as biomarkers of disease progression or diagnostic predictors. AD is a slowly progressive disorder and systemic changes in the blood are likely to be subtle and difficult to monitor. There are also technical limitations in assaying low abundant cytokines and many factors likely influence plasma immune markers, such as concomitant infection and inflammatory illness. Furthermore, many cytokines has been shown to display diurnal variation [25] and different handlings as well as storage of samples are known to affect the levels of many biomarkers. Therefore, standardization of pre-analytical procedures is vital to obtain reproducible results. To increase the possibility of successful reproduction of biomarker studies in the future the handling of samples should be carefully described, including data describing the time from venipuncture to minus 80 freezer storage, time of day that venipuncture was performed and if samples were collected fasting or non-fasting. Moreover, when using samples from different clinical centres all diagnostic groups (including controls) need to be obtained from each clinical centre in order to be able to investigate potential variations in biomarker levels between different clinical sites.

A limitation of this study is that the array-based method used is a potentially unreliable tool to disprove the original study by Ray et al, who also used a similar and non-validated method. However, in the present study we selected two cytokines for confirmation measurements with a standardized ELISA method, and again found no statistical differences between control and AD samples.

Our results indicate that multiplex platforms might be important for biomarker discovery, but validation of the results using new patient cohorts as well as other analytical techniques are vital. At least two patient-control cohorts with all important diagnostic groups present in each will likely be needed to verify obtained data. Importantly, recent data show that highly cited biomarker studies often report larger effect estimates than are reported in subsequent meta analyses [26]. This further strengthens our notion that validation is crucial in biomarker research.
